# Establishing Sexual Assault Care Centres in Belgium: health professionals’ role in the patient-centred care for victims of sexual violence

**DOI:** 10.1186/s12913-018-3608-6

**Published:** 2018-10-22

**Authors:** Anke Vandenberghe, Bavo Hendriks, Laura Peeters, Kristien Roelens, Ines Keygnaert

**Affiliations:** 1 International Centre for Reproductive Health (ICRH), Department of Uro-Gynaecology, Faculty of Medicine and Health Sciences, Ghent University, Corneel Heymanslaan 10, UZP114, 9000 Ghent, Belgium; 2 Department of Obstetrics and Gynaecology, Faculty of Medicine and Health Sciences, Ghent University, Ghent University Hospital, Corneel Heymanslaan 10, UZP3, 9000 Ghent, Belgium

**Keywords:** Sexual assault, Sexual violence, Sexual assault care Centre, Patient-centred care, Holistic care, Primary health care

## Abstract

**Background:**

Having ratified the Convention of Istanbul, the Belgian federal government commits itself to the foundation of Sexual Assault Care Centres (SACC). In the light of researching the feasibility of these centres, this study aimed to evaluate the care for victims of sexual violence (SV) in Belgian hospitals anno 2016 as well as to formulate recommendations for the intended model.

**Methods:**

Between April and October 2016, a questionnaire was distributed to 159 key health professionals active in 17 different hospitals attached to an AIDS Referral Centre. The survey covered four parts, i.e. the health professionals’ profile, their knowledge, attitude and practices, an assessment of the hospital’s policy and the caregivers’ opinion on the care for victims of SV and on the intended SACCs. Subsequently, a descriptive analysis using ‘IBM SPSS Statistics 23’ was performed.

**Results:**

A total of 60 key health professionals representing 15 different hospitals completed the questionnaire resulting in a response rate of 38%. The results showed a lack of knowledge and practical experience of caregivers’ regarding the care for SV victims. Approximately 30% of responders face personal or professional difficulties upon provision of care to victims of SV. Participants evaluate the current care as good, despite the limited psychosocial support, follow-up, insight for the needs of vulnerable groups and support for family, relatives and health professionals. Yet, the majority of health professionals appraise the SACCs as the best approach for both victims and caregivers.

**Conclusions:**

By introducing a SACC, the Belgian federal government aims to provide holistic and patient-centred care for victims of SV. Essential in patient-centred health care is an extensive and continuous education, training and supervision of health professionals concerning the care for victims, support for family, relatives and caregivers.

At the end and as a result of a participatory process with many professional experts as well as victims, a specific Belgian model, adjusted to the health care system anno 2016 was developed for piloting. The main challenges in establishing SACCs are situated at the institutional and policy level. Collaborating with other institutions and further research are herewith required.

**Electronic supplementary material:**

The online version of this article (10.1186/s12913-018-3608-6) contains supplementary material, which is available to authorized users.

## Background

The World Health Organisation’s (WHO) definition of sexual violence (SV) in 2015 is: “any sexual act that is perpetrated against someone’s will” committed “by any person regardless of their relationship to the victim, in any setting”. It includes, but is not limited to, rape, attempted rape and sexual slavery, as well as unwanted touching, threatened SV and verbal sexual harassment [1].

SV is a major public health problem [2], with a lifetime prevalence in European women of 5,2% committed by a non-partner and 25,4% by the victims (ex-)partner [3]. The Sexpert study in Flanders found a prevalence of 22,3% and 10,7% in case of respectively girls and boys. For adult women and men the percentages of lifetime victimization were respectively 13,8% and 2,4% [4]. A multi-level analysis in 10 European countries showed lifetime sexual victimisation rates of 20,4% and 10,1% for respectively Belgian young women and men aged 16 to 27 years [5]. The prevalence of SV in case of Lesbian, Gay, Bisexual and Transgender people (LGBT’s) is 31,7% to 41,1% and in case of migrants 56,6%, making them groups at risk [6–9]. However it remains impossible to exactly compare prevalence studies, due to differences in study designs, selection and response bias. Nevertheless since only 1 out of 10 victims report SV, the prevalence is strongly underestimated [10–12].

SV has important physical, reproductive and psychological implications for victims [13]. Many patients develop symptoms of functional somatic syndromes, posttraumatic stress disorder (PTSD), depression, substance abuse and despair [13, 14], in the context of facing stigmatization, rape myths and stereotypes [15]. In order to provide the needed support, international guidelines state that caregivers should recognise these symptoms and explore the patient’s history of SV [16, 17].

**Fig. 1:**
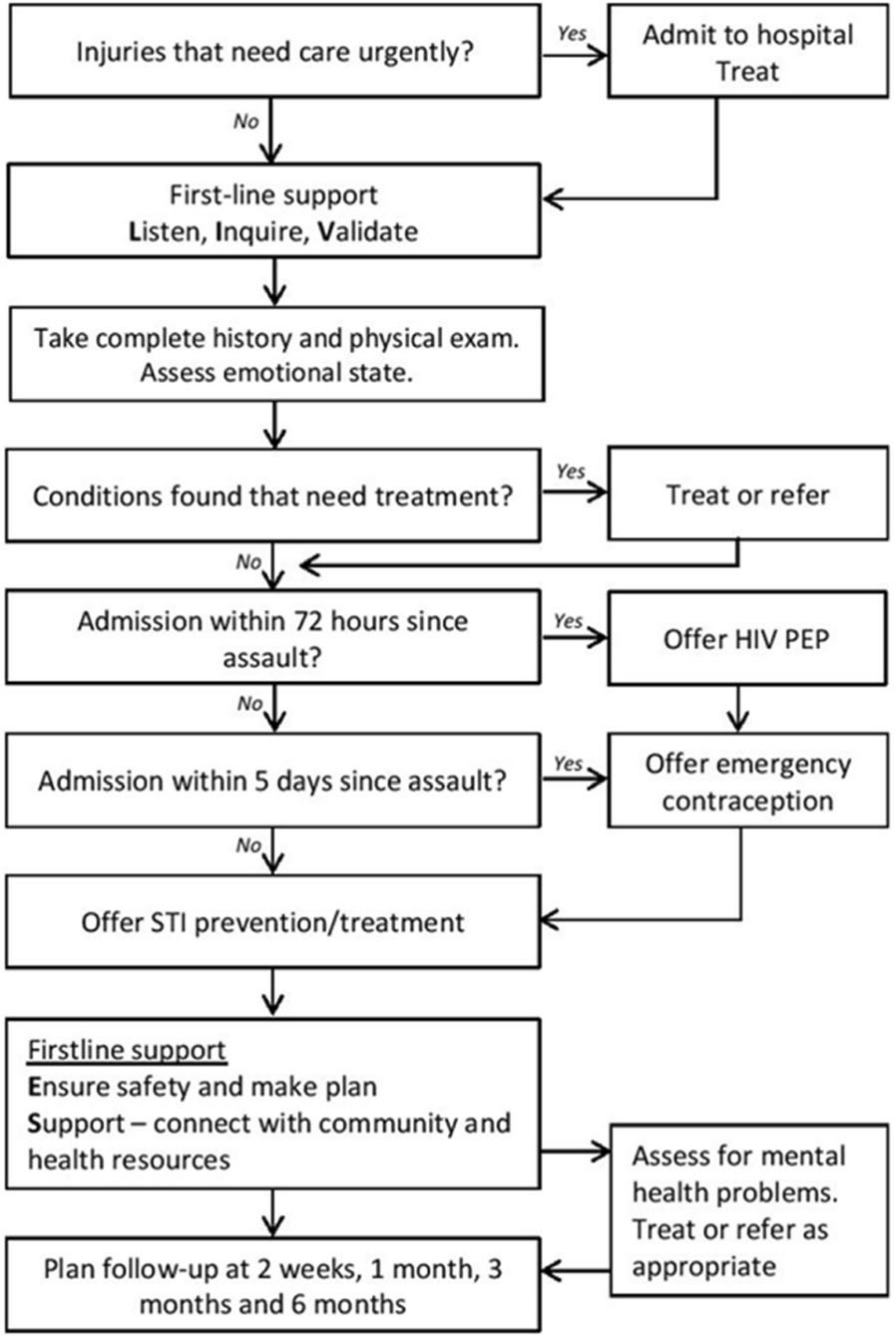
WHO’s recommended pathway for initial care after acute sexual violence [17]. Abbreviations: *WHO* World Health Organisation, *HIV* Human immunodeficiency virus, *PEP* Post-exposure prophylaxis, *STI* Sexually transmitted infection

Figure 1 illustrates the WHO’s recommended initial care after acute sexual assault [17]. Early presentation is crucial for the forensic examination, tests, proper treatment and referral within 72 h after the incident [11, 15]. Guidelines advice to conduct the forensic examination simultaneously with the physical examination, according to the anamnestic findings, at the victim’s pace and after receiving informed consent [15, 18, 19]. The required safety and privacy should be guaranteed [16, 20]. Depending on the victim’s wishes, a family member, relative or attendant should be able to offer support during the examination. The patient should never be fully undressed, while examinations and interviews should be reduced to a minimum where possible. Caregivers should give patients the opportunity to make an informed autonomous treatment choice and respect the choice made [15, 16]. Every victim also needs appropriate support from family, relatives and health professionals with regular follow-up during the first 1–3 months [17]. At follow-up consultations patients should be asked about treatment difficulties, their personal lives, their emotional wellbeing and their concerns [21].

According to the WHO, a holistic approach of health care focuses not only on the patient’s physical, emotional and social needs but also on their past, future and broader context, requiring a strong collaboration among different stakeholders [22].

By integrating the perspective, needs and preferences of patients, patient-centred care tries to focus on the wellbeing of the victim [17, 21]. After SV many victims have lost control and autonomy of their lives. In order to regain this control and autonomy, it is required to provide a safe environment and thorough information. To this end, caregivers should acquire appropriate knowledge, attitudes and practice in caring for victims of SV [21, 23].

Furthermore, the access to health care plays a central role in a patient-centred approach, which can be evaluated using 5 dimensions, i.e. accessibility, acceptability, availability, affordability and appropriateness [24].

As part of the broader context of the victim, it is desirable for caregivers to support the family and relatives as well [17, 21, 25]. Family and relatives can be affected in different ways. First, because SV to a loved one is a trauma for themselves, affecting their emotional wellbeing as well. Secondly they might be a victim or witness of SV themselves and need psychosocial support [25, 26].

The intended model for the foundation of a Sexual Assault Care Centre (SACC) in Belgium builds on existing Sexual Assault Referral Centre (SARC), Sexual Assault Nurse Examiner (SANE), Sexual Assault Treatment Unit (SATU) and Sexual Assault Referral Team (SART) models [9, 27–30]. In order to give holistic and patient-centred care, the model aims to provide acute and follow-up care at one place provided by one central health professional supported by a team of specialist professionals, as recommended by the WHO [15]. The SACC will be located nearby an emergency service on hospital grounds with a separate access and a guaranteed 24/7 accessibility.

During the first contact in the SACC, a forensic nurse or SANE will take care of the acute forensic, medical and psychosocial support of the victim. Other responsibilities of the forensic nurse are online and telephonic assistance, registration and raising awareness about SV.

After the acute support, the case manager is the single point of contact and is responsible for adequate follow-up, referral and the cooperation with different stakeholders in the care for SV victims.

A team of professionals consisting of a gynaecologist, an urologist, an infectious disease specialist, a paediatrician, a forensic physician and a psychiatrist will provide support to the forensic nurse and the case manager if needed.

**Fig. 2:**
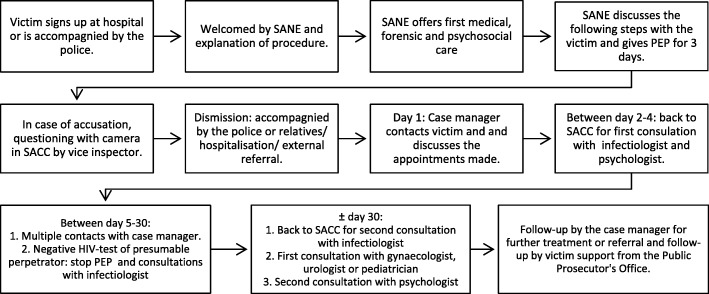
Intended pathway for initial care after acute sexual assault in a Belgian SACC. Abbreviations: *SACC* Sexual Assault Care Centre, *SANE* Sexual Assault Nurse Examiner, *PEP* Post-exposure prophylaxis, *HIV* Human immunodeficiency virus

During the first month post assault the case manager will contact the victim at least 4 times, starting at day 1 after discharge. Furthermore at least 2 appointments with the SACC’s psychologist will be arranged. A total of 12 to 20 free appointments with a qualified psychologist are provided depending on the patient’s wishes and psychosocial assessment. When indicated 1 appointment at the AIDS Referral Centre (ARC), gynaecologist, urologist or paediatrician will be scheduled. A schematic overview clarifies the initial care after acute sexual assault in the intended SACC (see Fig. 2).

In order to guarantee specialised care, every stakeholder attached to the SACC will receive a common training in the care for victims of SV, followed by a function-specific training for the forensic nurse, case manager and psychologist [31].

Having ratified the convention of Istanbul, the Belgian federal government intended to explore the feasibility of raising SACCs in the Belgian health and judiciary system [32, 33]. This project aimed to assess the care for victims of SV in Belgium anno 2016 and evaluate the transition to the eligible approach.

In the context of this project, this study focused on the evaluation of the health care for victims of SV in Belgian hospitals anno 2016 and more specifically on the role of caregivers in establishing a patient-centred care aiming to formulate recommendations for the intended model.

## Methods

### Survey design

An extensive review of the WHO [1, 15–17], the National Institute for Health and Care Excellence (NICE) [25, 34] and the Centers for Disease Control and Prevention (CDC) guidelines [35], good practices in recent literature [13, 14, 18–20] and existing SACC models from The Netherlands, the United Kingdom, Ireland, the United States, Denmark, Norway and Sweden [9, 27, 29, 30, 36–41], was conducted as a preparation for the experimental phase and discussion. In cooperation with a team of experts a Knowledge Attitude and Practice (KAP) survey was developed between March and April 2016, based on a prior study in East Flanders [42], recent literature and guidelines.

The first part scrutinised the caregiver’s profile. Secondly their knowledge of, experience with and attitude towards SV was assessed with answers scored by a five-point Likert scale. In order to evaluate their knowledge, the participants were given different statements regarding the evidence-based prevalence of SV [2–4, 6–10]. Aiming to assess the health professionals’ experience with SV victims, the survey investigated their estimation of the number of victims reporting SV, the number of victims they have supported and their presumption of SV among other patients. Regarding their attitude towards SV, the questionnaire evaluated their opinion on discussing SV with patients or victims, testifying in a lawsuit and their personal and professional consequences upon provision of care for victims of SV.

The third part explored the content of their hospital’s general policy against violence and their care for victims. If available, the participants were asked to specify the subject, the target audience, the gender sensitivity and the mode of implementation. Furthermore the survey explored the existence of violence coordination, training and support of health professionals. Regarding the care for victims of SV, the questionnaire scrutinised the accessibility of care, the privacy and safety of patients, the availability and use of equipment, tests, a Sexual Aggression Set (SAS) and treatment options, the approach to data registration, psychosocial support and follow-up.

Finally, their appreciation of the support for victims of SV, the cooperation with other instances and the foundation of SACCs was explored and scored by a five-point Likert scale as well.

### Data collection

Adequate Human Immuno-deficiency Virus (HIV) post-exposure prophylaxis (PEP) and follow-up care are crucial in the delivery of holistic health care for victims of SV. Given that the Belgian law only foresees reimbursement if treated in an ARC, the Belgian Sexual Assault Referral Centre (B-SARC) feasibility study included solely hospitals with an ARC(−hub). The management, medical and nursing directors of 17 Belgian hospitals were invited to participate by the Belgian federal Secretary of State for Equal Opportunities. They were asked to identify and to transfer the contact details of the health professionals most involved in the care for victims of SV active in the department of emergency medicine, gynaecology, urology, psychiatry, paediatrics, the ARC and social service. The principal researcher then invited each key health professional personally by post or mail to complete a printed survey. The questionnaire was available in Dutch and French, covered four parts and included most closed, semi-open and a few open questions. Participants could reply in writing or in a recorded semi-structured face-to-face or phone interview and were asked to sign an informed consent form after reading the accompanying information letter.

### Data analysis

All interviews were transcribed *ad verbatim*. Two researchers conducted a global descriptive analysis, both at service and hospital level and coded the open questions’ responses using the Framework Method [43]. The different responses were then integrated in IBM SPSS Statistics 23, after comparison, categorisation and approval by the principal investigator, resulting in 403 variables. An estimation of the health support for victims of SV anno 2016 was based on the majority’s responses, taking the different services and hospitals into account. Subsequently, we compared the findings to the recommendations from international guidelines [1, 15–17, 25, 34, 35], good practices in recent literature [13, 14, 18–20] and existing SACC models [9, 27, 29, 36–41].

### Ethics approval

Ethical approval (EC/2016/0499) was received from the Ethical Committee of the Ghent University Hospital and a Belgian registration number (B670201628242) was granted.

## Results

### Participants’ profile

Of the 159 contacted health professionals 60 completed a questionnaire and signed an informed consent form, matching a response rate (RR) of 38% (see Additional file 1). Two caregivers refused to participate, four stated to be short of time or to have a lack of experience and 93 never responded by 1 October 2016. Eight of the 60 participants preferred to conduct a recorded semi-structured face-to-face interview. No one replied with a phone interview. The services gynaecology, emergency, paediatrics and ARC are well represented as illustrated in Table 1.

**Table 1:** Responding Belgian experts’ demographics

		n			n	RR
Sex	Male	22	Service	Gynaecology/maternity	11	0,46
	Female	38		Emergency	10	0,43
Age	25–29 years	1		Psychosocial service	7	0,32
	30–39 years	15		Psychiatry	5	0,24
	40–49 years	23		Paediatrics	11	0,50
	50+	21		Urology/ gastroenterology	5	0,22
Function	Social assistant	4		AIDS Referral Centre	10	0,43
	Psychologist	3		Forensic medicine	1	1,00
	Head nurse	4	Period employed at service	less than 2 years	5	
	Physician	22		2–5 years	7	
	Head of service	22		6–10 years	13	
	Head of clinic	5		11–15 years	11	
				16–20 years	9	
				more than 20 years	13	

‘n’: frequency of a certain variable in the sample; ‘RR’: response rate

The participating caregivers are mainly female and physicians. Others are head nurses, psychologists or social assistants. Half of the participants are already being employed at their service for over 11 years.

### Knowledge of the prevalence of sexual violence

Of all respondents 72% completely agreed that SV is a major public health problem worldwide. However they underestimated the magnitude of the problem of SV in Belgium. Only the number of adult female victims was correctly assessed by 58% of the participants. Caregivers active in an ARC, the emergency and the gynaecology department showed more accurate estimates of the SV prevalence rates (Table 2).

**Table 2:** Responding Belgian experts’ knowledge of the prevalence of sexual violence

	Completely disagree		Disagree		I don’t know		Agree		Completely agree		Total	
	n	P	n	P	n	P	n	P	n	P	N	P
SV is a major public health problem worldwide in Belgium	0	0%	0	0%	0	0%	17	28%	43	72%	60	100%
	0	0%	3	5%	2	3%	38	63%	17	28%	60	100%
Prevalence of sexual violence in Belgium:												
25% of girls (<18y)	4	7%	10	17%	26	43%	13	22%	7	12%	60	100%
15% of women (>18y)	1	2%	3	5%	21	36%	27	46%	7	12%	60	100%
10% of boys (<18y)	0	0%	6	10%	35	58%	13	22%	6	10%	60	100%
5% of men (>18y)	1	2%	1	2%	33	55%	22	37%	3	5%	60	100%
30% of transgender	0	0%	1	2%	35	58%	20	33%	4	7%	60	100%
40% of lesbians, gays or bisexuals	0	0%	5	8%	37	62%	14	23%	4	7%	60	100%
40% of migrants	0	0%	6	10%	37	62%	14	23%	3	5%	60	100%
Irregular migrants are more vulnerable to SV	0	0%	2	3%	6	10%	36	60%	16	27%	60	100%
Physical/mental disabled people are more vulnerable to SV	0	0%	0	0%	7	12%	31	52%	22	37%	60	100%

‘n’: frequency of a certain variable in the sample; ‘N’: sample size; ‘P’: observed percentage of a certain variable in the sample

Abbreviations: *SV* Sexual violence

### Experience with victims of sexual violence

When exploring how many victims reported their incident of SV to the hospital in the last 12 months, the answers varied widely within the different hospitals and services. Almost one fourth (*n* = 13; *N* = 60) claimed that 50 or more patients reported to their hospital and 65% (*n* = 39; *N* = 60) took care of less than 10 victims in the past year. Nevertheless, one third (*n* = 20; *N* = 60) stated to be unaware how many victims reported to their hospital.

Because the care for victims of SV goes beyond acute support and many patients never report a history of SV, the caregivers’ presumption of SV among other patients was explored. Approximately half of the participants (*n* = 33; *N* = 59) indicated to suspect a history of SV in less than 10 patients in the past 12 months. Of the participants able to assess their suspicion of a history of SV among their patients (*n* = 51; *N* = 58), 33 caregivers always addressed this presumption.

Table 3 illustrates the evaluation of the caregivers’ attitude towards discussing a history of SV with their patients. The majority seemed to have no problem discussing SV and claimed to be motivated to counter SV, as well as to possess the necessary expertise or to feel responsible to help identifying victims of SV. The existing stigma, language barriers, the presence of parents as well as the lack of time, of training, of qualified staff or of financial resources are reported to be a threshold in discussing SV and identifying SV victims in daily practice.

**Table 3:** Responding Belgian experts’ opinion towards discussing sexual violence with patients

	Completely disagree		Disagree		I don’t know		Agree		Completely agree		Total	
	n	P	n	P	n	P	n	P	n	P	N	P
No problem, skilled enough	0	0%	11	19%	2	4%	33	58%	11	19%	57	100%
No problem, experienced enough	2	4%	13	23%	1	2%	29	51%	12	21%	57	100%
Not obvious, not acceptable for patients	8	14%	23	41%	4	7%	19	34%	2	4%	56	100%
Not obvious regarding current organisation	11	20%	25	45%	0	0%	14	25%	6	11%	56	100%

‘n’: frequency of a certain variable in the sample; ‘N’: sample size; ‘P’: observed percentage of a certain variable in the sample

### Support for victims of sexual violence^1^

Physicians are generally responsible for the acute care for adult victims of SV, particularly gynaecologists (*n* = 34; *N* = 56) followed by urologists (*n* = 29; *N* = 54), emergency physicians (*n* = 17; *N* = 53) and forensic physicians (*n* = 12; *N* = 53). In case of children, participants stated that beside gynaecologists (*n* = 34; *N* = 57) also paediatricians (*n* = 29; *N* = 52) or forensic physicians (*n* = 18; *N* = 52) can offer the required support. Nurses mainly have a supportive function in the acute and long-term care for SV victims, as commissioned by the Belgian law system [44]. However, answers varied widely within different services of a hospital, indicating the absence of a standard protocol.

A thorough physical examination and a standard SAS, containing all elements for forensic evidence collection, are performed upon police audit. The use of tests, i.e. on gonorrhoea (*n* = 7, *N* = 41), on chlamydia (*n* = 7, *N* = 41), on trichomonas vaginalis and bacterial vaginosis (*n* = 9, *N* = 49), on syphilis (*n* = 8, *N* = 40), on HIV (*n* = 7, *N* = 40), on HPV (*n* = 9, *N* = 42) and a pregnancy test (*n* = 7, *N* = 39), appeared to be unclear to approximately 20% of the participants. Moreover, the results showed a limited knowledge regarding the provision and use of the morning after pill (n_a_ = 13, N_a_ = 53; n_u_ = 13, N_u_ = 46), the HIV PEP for 3 days (n_a_ = 14, N_a_ = 55; n_u_ = 14, N_u_ = 43) and 1 month (n_a_ = 16, N_a_ = 55; n_u_ = 16, N_u_ = 45), the vaccination for hepatitis B (n_a_ = 13, N_a_ = 55; n_u_ = 14, N_u_ = 46) and tetanus (n_a_ = 13, N_a_ = 55; n_u_ = 13, N_u_ = 45). In some of the participating hospitals a social assistant is responsible for the acute psychosocial support (*n* = 7; *N* = 15). Concerning the different options of psychosocial support, i.e. watchful waiting (*n* = 19, *N* = 50), psychosocial assistance (*n* = 10, *N* = 57), risk-analysis for PTSS (*n* = 22, *N* = 47), PTSS-analysis (*n* = 22, *N* = 50) and EMDR (*n* = 23, *N* = 52), 18% to 47% stated to be unaware. If provided, psychosocial follow-up consists of watchful waiting (*n* = 26; *N* = 50) and minimal psychosocial assistance (*n* = 45; *N* = 57). The treating physician, emergency physician, ARC, social assistant or gynaecologist is responsible for follow-up, nevertheless according to 29 health care providers (*N* = 53) there is no control of follow-up. Participants were well informed on the accessibility, privacy, equipment and follow-up in their hospital. However, they were less familiar with the financial cost for the patient (*n* = 24, *N* = 42), the process of a physical and forensic examination (*P* = 25% to 30%, *N* = 53 to 56) and the availability of a protocol for data registration (*n* = 17, *N* = 60).

**Fig. 3:**
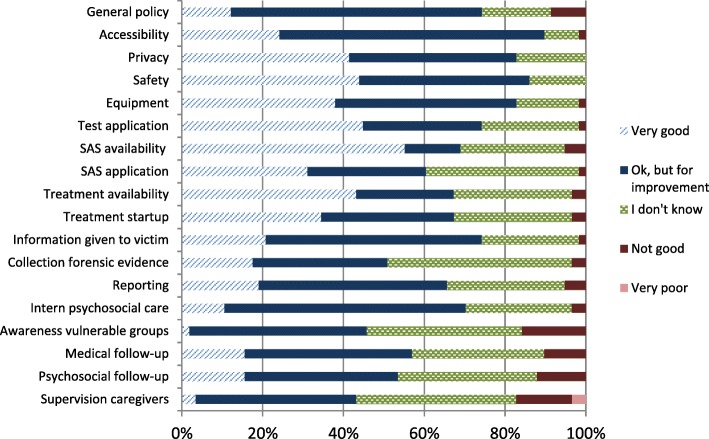
Responding Belgian experts’ evaluation of the hospital’s policy. Sample size = 58 in accordance with a percentage of 100%. Abbreviations: *SAS* Sexual Aggression Set

When assessing their hospital’s general policy on SV, 43 participants evaluated it well to very well (*P* = 74%; *N* = 58). Only caregivers working in the urology department reported to have insufficient knowledge to be able to assess their general policy (*n* = 3; *N* = 5). Furthermore a majority of 82% to 90% (*N* = 58) considered the accessibility, privacy, safety (*N* = 57) and equipment as good to very good. Only the awareness of the needs of vulnerable groups (*P* = 16%; *N* = 57) and the supervision of caregivers themselves (*P* = 17%; *N* = 58) are experienced as not good to inferior (see Fig. 3).

### Impact on the well-being of caregivers

The care of victims of SV can negatively impact caregivers’ personal and professional lives. Overall 35% (*n* = 14; *N* = 40) of health care providers encounter emotional issues. They seem to identify themselves to the victim, have a personal history of violence or feel depressed. Other participants are however motivated to combat SV or have an adequate coping strategy and sufficient social support. When caring for victims, 29% (*n* = 11; *N* = 38) faces practical problems, e.g. too late referral in order to still receive adequate treatment, too much administration and lack of time, experience, teamwork, reflection and follow-up. A few responders (*n* = 4; *N* = 38) are satisfied to be able to help victims of SV. Some caregivers (*n* = 14; *N* = 38) experience no impact on their professional lives due to a clear protocol, a multidisciplinary team and great flexibility.

Despite the repercussion on their wellbeing, 25 caregivers (*N* = 51) indicate not being provided any supervision or support while 23 were not trained on SV. Education concerning SV in vulnerable groups, i.e. LGBT’s (*n* = 2), migrants (*n* = 4) and disabled people (*n* = 1), is nearly non-existent (*N* = 51). The majority does discuss cases of violence with the team or department (*n* = 41; *N* = 51) and has safety measures at their emergency department (*n* = 42; *N* = 59).

### Contribution of family support

The questionnaire inquired the availability of support for family and relatives. As for the protocol on SV taking every other person involved into consideration, the answers varied widely, which makes distinction between protocol and practice difficult. However, in case of child abuse, caregivers seemed to be more attentive to the context of the child. According to the majority (*n* = 52; *N* = 60) there is a waiting room available for the family and relatives of victims at the emergency, paediatric or gynaecology department. If requested by the victim, confidential persons have the opportunity to attend the examination (*n* = 50; *N* = 59), though limited by number (*n* = 31; *N* = 59) and age (*n* = 23; *N* = 59). Merely a minority of caregivers (*n* = 18; *N* = 55) indicated to offer psychosocial support for family and relatives of the victim.

### Appreciation of SACCs

Exploring the participants’ acquaintance about SACCs, almost half (*n* = 25; *N* = 58) stated to be unaware of its existence. The caregivers’ attitudes towards the foundation of SACCs are illustrated in Table 4. They appraise SACCs as the best approach for patients and caregivers. Only the fear for prosecution remains a possible impediment, e.g. for illegal residence, ongoing legal proceedings, open warrants and prosecution of the victim’s partner. Some suggest that it will benefit the accessibility and quality of health care and lower the pressure on the emergency department. Although evaluated as necessary, approximately 20% of caregivers stated to be unaware or unable to give psychosocial care (*n* = 13; *N* = 59) and follow-up (*n* = 15; *N* = 59). Other mentioned sufficient SACCs, staff, materials, financial resources, translation facilities and raising awareness as fundamental to improve the accessibility and quality of care. However, some are uncertain if their hospital would modify their protocol to the national policy (*n* = 24; *N* = 59). The majority would communicate information about the assault with other hospitals (*n* = 43; *N* = 57) or the general practitioner (*n* = 47; *N* = 58), though only with the patient’s permission. In order to succeed, good communication of the hospitals raising a SACC is essential (*n* = 56; *N* = 58).

**Table 4:** Responding Belgian experts’ opinion of a SACC as the ideal approach for patients and caregivers

	Completely disagree		Disagree		I don’t know		Agree		Completely agree		Total	
	n	P	n	P	n	P	n	P	n	P	N	P
For patients												
No precious time lost	3	5%	0	0%	1	2%	13	22%	41	71%	58	100%
Patient doesn’t have to tell the history repeatedly	2	3%	1	2%	0	0%	15	26%	40	69%	58	100%
Patient gets specialised care	0	0%	1	2%	0	0%	11	19%	46	79%	58	100%
Patient receives long-time follow-up	2	4%	1	2%	2	4%	22	39%	30	53%	57	100%
Patient doesn’t have to fear prosecution	3	5%	1	2%	7	12%	15	26%	31	54%	57	100%
For caregivers												
Ability to focus on this issue	1	2%	1	2%	1	2%	20	34%	35	60%	58	100%
Ability to work in a multidisciplinary team	0	0%	1	2%	0	0%	15	26%	42	72%	58	100%
No pressure of patients waiting	1	2%	2	4%	5	9%	19	33%	30	53%	57	100%
Adjusted financial prospects of the management	1	2%	1	2%	17	31%	12	22%	24	44%	55	100%

‘N’: sample size; ‘n’: frequency of a certain variable in the sample; ‘P’: observed percentage of a certain variable in the sample

Abbreviations: *SACC* Sexual Assault Care Centre

## Discussion

According to the results, many different health professionals are involved in the care for victims of SV, psychosocial care is limited and follow-up is inadequately organised resulting in many patients being lost to follow-up. Moreover the support for SV victims mainly focuses on medico-legal concerns and only offers forensic examination upon police audit. These findings reflect the fragmented health care system and a lack of accessibility.

The participants’ opinion regarding the health care for SV victims in Belgium is good to very good. However, the awareness for the needs of vulnerable groups, the supervision for health professionals, the psychosocial support for and the follow-up of victims still requires improvement. Despite the lack of supervision and training, health professionals feel comfortable in discussing SV with their patients, reflected in the majority addressing a presumption of SV among their patients. Although some (incorrectly) believe patients do not accept screening without their consent and feel limited due to practical issues. These findings are similar to previous research in the Flemish region of Belgium [42, 45, 46].

Together with a growing body of literature, a more horizontal, holistic and patient-centred care in Belgium endorses itself [1, 9, 17, 28]. In the proposed model forensic nurses will provide acute medical, forensic and psychosocial support. The case manager will guarantee patients’ follow-up in order to provide a more organised and continuous health care. With the support of a team of professionals, the maintenance of specialised care is assured. Furthermore, the victim will receive at least 2 contacts with the SACC’s psychologist during the first month with a maximum of 12 to 20 free appointments according to the patient’s wellbeing. Therefore, the implementation of a SACC will additionally contribute to a better psychosocial support and a more patient-centred and less medico-legal focused approach.

In the light of providing holistic care, health professionals should have sufficient knowledge, skills and attitudes concerning SV [21, 23, 47]. However, this study pointed out an insufficient knowledge among caregivers regarding the prevalence of and support for victims of SV, varying concordantly with their expertise area.

These findings are in accordance with their limited experience with SV victims and low presumption of SV among their patients. Furthermore the results showed a more supportive function of nurses, while different physicians perform a leading function in the care for victims of SV. In contradiction, physicians state to be provided limited to no education or training on care provision for victims of SV. Health professionals active in a SACC however play a major role in a holistic approach [22]. Therefore, a thorough education and training of every health professional attached to a SACC will be conducted.

Reaching a horizontal, holistic and patient-centred care for victims of SV, goes beyond the foundation of SACCs. A good collaboration between different stakeholders in the multidisciplinary care for victims of SV is fundamental [22]. Therefore, the principal researcher organised simultaneous researches with the police and law enforcement.

In order to delineate the victims’ needs and concerns, a simultaneous qualitative study with former victims of SV was additionally conducted.

### Recommendations

As described above, a SACC offers opportunities to tackle many challenges in providing a more holistic and patient-centred health care. Its implementation however faces some impediments.

As the Belgian system only allows a physician to conduct a forensic examination and treatment initiation [44], it requires legal permission to let a forensic nurse provide acute medical, forensic and psychosocial support.

Due to the diversity of the approach within different hospitals and services, there is a lack of accessibility and equality in the provision of health care for victims of SV. To this end, the procurement of a nationwide protocol and good collaboration with other institutions is essential to an equal, continuous and accessible health care. Furthermore in order to raise the accessibility and the awareness among the population, the Belgian government should conduct an extensive communication campaign.

Moreover this study included only key health professionals, meaning these results are positively distorted in comparison with other caregivers. Knowing only the minority of victims seek professional help despite the many consequences [10–12], every health professional, regardless of their relationship to a SACC, should receive education and training in the care for SV victims [48]. In addition a SACC could provide further education and training for health care providers, beside the necessity to include the care for victims of SV in mainstream medical and paramedical curricula.

The care for family and relatives of victims of SV is part of a holistic health care approach. A waiting room and the possibility to attend the examination at patient’s request are feasible in every participating hospital as recommended by the WHO [1, 17]. However, the support for family and relatives of SV victims seems to be minimal to absent. The provided psychosocial care and attention in protocols is restricted despite recent WHO and NICE guidelines [17, 25]. Nevertheless the provision of information, professional support and adequate referral will not only contribute to an improved wellbeing of victims and their family or relatives but also to a greater accessibility of health care [26].

Because health professionals play an important role in the recovery of SV victims, they are also part of the victim’s broader context. This means attention should go to supervision of health care providers, as their wellbeing and coping mechanisms could affect the quality and accessibility of health care [49]. Nevertheless, caregivers indicate this supervision as a point of improvement. Moreover participants state to experience personal and professional complications in providing care to SV victims, confirming recent literature [50]. Noteworthy is the amount of missing data when evaluating health professionals’ wellbeing, possibly strengthening the stigma associated, the small awareness and the limited support for caregivers of SV victims. Consequently, the support for caregivers is perceived as poor and could be improved by informing, coordinating, managing, supervising and raising awareness of different actors in caring for SV victims.

In establishing SACCs, the dual role of forensic nurses in providing emotional support and collecting evidence needs to be taken into consideration [47, 51]. Besides the positive and constructive feedback of patients and the support for professionals, a good interaction with colleagues can contribute to a greater wellbeing of health professionals [52].

Finally, further research should go to effective measures for support for health professionals and family or relatives of SV victims. As general practitioners can play a major role in the health care for victims of SV [53, 54], it is absolutely recommended to conduct such a research in a second phase. Likewise, the experiences and knowledge of every health professional and the contribution of education and training need to be investigated.

### Limitations

With the high prevalence of SV and the minority of victims seeking professional help, every health professional will be confronted with SV victims. However, this research included solely key health professionals identified by the management and the medical director working in a hospital attached to an ARC. Therefore, the data-collection contributes to a selection bias due to the exclusion of other caregivers having (in-)direct contact with victims of SV. Moreover, the response rate for this study is low with only four stating to have a lack of time or experience. The low response rate is possibly a consequence of the previous mentioned selection bias, the method of data-collection, the high work load and lack of time of physicians in Belgium and the extent of the questionnaire. Due to this selection bias, the limited study sample and the low representation of some services, the description of the Belgian health care for victims of SV anno 2016 might not be plenary.

With the general practitioner playing a central role in primary health care [22], they will encounter victims of SV. Nevertheless this study did not include general practitioners because within the Belgian health system anno 2016, victims of acute SV tend to report to a hospital instead of their general practitioner. Therefore, the Belgian federal government chose to start the implementation of SACCs within a hospital-based environment.

Furthermore health care providers could reply in writing or in a recorded semi-structured face-to-face or phone interview in order to increase the accessibility to participate. This can contribute to the differences in the results. Nevertheless only a minority of health care providers preferred to conduct a face-to-face interview.

In the end, this research however brings a first extensive analysis of the care for victims of SV in Belgium commissioned by the Secretary of State for Equal Opportunities. More important, this study contributes to a greater project aiming to provide holistic and patient-centred care for victims of SV by establishing SACCs in Belgium.

## Conclusions

The high prevalence of SV, the fragmented health care, the lack of accessibility and growing evidence for a holistic and patient-centred approach favour the foundation of SACCs in Belgium, by introducing a forensic nurse for acute support and a case manager for follow-up located in one setting and supported by a professional team. Essential in providing patient-centred health care is an extensive and continuous education, training and supervision of caregivers working in SACCs. Furthermore, support for family, relatives and caregivers should be taken into account. The main challenges in establishing SACCs in Belgium are situated at institutional and policy level.

Imperative to improve access to health care is continuous education of every health professional concerning the care for victims of SV in combination with a good collaboration with other institutions e.g. the police, law enforcement, hospitals, support groups and general practitioners. Further research on effective support and training measures, general practitioners’ role and the KAP of every caregiver is needed.

At the end and as a result of a participatory process with many professional experts as well as victims, a specific Belgian SACC model, adjusted to the health care system anno 2016 was developed for piloting.

## Additional file

Additional file 1: Flow diagram data collection. Schematic overview of the method of data collection with indicated sample size and frequency of each contacted service. ‘N’: sample size; ‘n’: frequency of a certain variable in the sample. Abbreviations: *ARC* AIDS Referral Centre. (DOCX 35 kb)

## Abbreviations

ARC: AIDS Referral Centre
B-SARC: Belgian Sexual Assault Referral Centre
CDC: Centers for Disease Control and prevention
HIV: Human Immuno-deficiency Virus
KAP: Knowledge, Attitude and Practice
LGBT’s: Lesbian, gay, bisexual and transgender people
NICE: National Institute for Health and Care Excellence
PEP: Post-Exposure prophylaxis
PTSD: Posttraumatic stress disorder
SACC: Sexual Assault Care Centre
SANE: Sexual Assault Nurse Examiner
SARC: Sexual Assault Referral Centre
SART: Sexual Assault Referral Team
SAS: Sexual Aggression Set
SATU: Sexual Assault Treatment Unit
SV: Sexual Violence
WHO: World Health Organisation

## Footnotes

1 ‘n_a_/N_a_’: variable frequency/sample size regarding the availability of a certain test/treatment ‘n_u_/N_u_’: variable frequency/sample size regarding the use of a certain test/treatment
