# Supplementary material for: Establishing Sexual Assault Care Centres in Belgium: health professionals’ role in the patient-centred care for victims of sexual violence
Source: BMC Health Serv Res. 2018 Oct 22;18:807. doi: 10.1186/s12913-018-3608-6 (PMC6196455; doi:10.1186/s12913-018-3608-6)
Supplement: Supplementary file 1 — Flow diagram data collection. Schematic overview of the method of data collection with indicated sample size and frequency of each contacted service. ‘N’: sample size; ‘n’: frequency of a certain variable in the sample. Abbreviations: ARC AIDS Referral Centre. (DOCX 35 kb) [file 12913_2018_3608_MOESM1_ESM.docx]

Selection of hospitals attached to an AIDS Referral Centre (ARC) and disposing of an emergency service, a social service, a gynaecology, urology, paediatric and psychiatric department

(N=17)

Contact of the management, medical and nursing directors

Identification of key health professionals in the care for victims of sexual violence

Contacted health professionals

(N=159)

Non-participating health professionals:

(N=99)

- ARC (n=13)
- Emergency service (n=13)
- Social service (n=15)
- Gynaecology (n=13)
- Urology (n=18)
- Paediatric department (n=11)
- Psychiatric department (n=16)

Reasons for not participating:

- Refusal (n=2)
- Lack of time (n=2)
- Lack of experience (n=2)
- Non-responding (n=93)

Participating health professionals

(N=60)

- ARC (n=10)
- Emergency service (n=10)
- Social service (n=7)
- Gynaecology (n=11)
- Urology (n=5)
- Paediatric department (n=11)
- Psychiatric department (n=5)
- Forensic department (n=1)
